# Prescribing Time in Nature for Human Health and Well-Being: Study Protocol for Tailored Park Prescriptions

**DOI:** 10.3389/fdgth.2022.932533

**Published:** 2022-07-19

**Authors:** Courtney L. Schultz, Jason N. Bocarro, J. Aaron Hipp, Gary J. Bennett, Myron F. Floyd

**Affiliations:** ^1^Health & Technology Partners, Milwaukee, WI, United States; ^2^Department of Parks, Recreation & Tourism Management, College of Natural Resources, NC State University, Raleigh, NC, United States; ^3^Global Health Institute, Duke University, Durham, NC, United States

**Keywords:** park prescriptions, nature-based health interventions, nature's contribution to people (NCP), primary health care, outdoor physical activity, eHealth, nature as medicine

## Abstract

**Background:**

eHealth technologies offer an efficient method to integrate park prescriptions into clinical practice by primary health care (PHC) providers to help patients improve their health *via* tailored, nature-based health behavior interventions. This paper describes the protocol of the GoalRx Prescription Intervention (GPI) which was designed to leverage community resources to provide tailored park prescriptions for PHC patients.

**Methods:**

The GPI study was designed as a 3-arm, multi-site observational study. We enrolled low-income, rural adults either at-risk of or living with hypertension or diabetes (*n* = 75) from Federally Qualified Health Centers (FQHC) in two counties in North Carolina, USA into the 3-month intervention. Eligible participants self-selected to receive (1) a tailored park prescription intervention; (2) a tailored home/indoor PA prescription intervention; or (3) a healthy eating prescription (with no PA prescription beyond standard PA counseling advice that is already routinely provided in PHC) as the comparison group. The GPI app paired patient health data from the electronic health record with stated patient preferences and triggered app-integrated SMS motivation and compliance messaging directly to the patient. Patients were assessed at baseline and at a 3-month follow-up upon the completion of the intervention. The primary outcome (mean difference in weekly physical activity from baseline (T0) to post-intervention (T1) as measured by the Fitbit Flex 2) was assessed at 3 months. Secondary outcomes included assessment of the relationship between the intervention and biological markers of health, including body mass index (BMI), systolic and diastolic blood pressure, HbA1c or available glucose test (if applicable), and a depression screen score using the Patient Health Questionnaire 9. Secondary outcomes also included the total number of SMS messages sent, number of SMS messages responded to, number of SMS messages ignored, and opt-out rate.

**Discussion:**

The goal was to create a protocol utilizing eHealth technologies that addressed the specific needs of rural low-income communities and fit into the natural rhythms and processes of the selected FQHC clinics in North Carolina. This protocol offered a higher standard of health care by connecting patients to their PHC teams and increasing patient motivation to make longer-lasting health behavior changes.

## Introduction

Nature has been shown to have positive impacts on physical, psychological, and social well-being (1–3). Since 1948, the World Health Organization has defined health as “a state of complete physical, mental, and social well-being and not merely the absence of disease or infirmity” (4). This definition of health shifted from an outdated disease-centric model to a salutogenic approach in which our ability to promote health is reliant upon human activity and the physical and biological environment. Despite this long-standing connection, in the U.S., a pathogenetic approach to health has traditionally focused on mechanisms that cause disease, and the role of primary health care (PHC) has been to treat illness rather than promote health (5). An increasing burden of non-communicable diseases and the associate cost of health conditions related to physical inactivity is driving the transformation of PHC toward integrating disease prevention alongside the treatment of disease (6). Over a lifetime, personal behaviors have the greatest influence on health compared to other health determinants. In the U.S., nearly 90% of personal health care expenditures are spent on direct care, leaving limited resources for changing health behaviors (7). More than $117 billion in annual healthcare costs in the United States are the result of insufficient physical activity levels (8). Current access to care and quality of care are insufficient for health; as a result, there is a recognition of the need for more social determinants of health interventions within PHC.

The integration of nature-based physical activity counseling and prescriptions into clinical practice by PHC providers can help patients improve their health *via* local, culturally appropriate, health behavior interventions (9–11). Physical activity counseling in PHC significantly increases physical activity (11) and is most successful when done through tailored approaches (12). A systematic review of the effectiveness of physical activity interventions found evidence that the uptake of physical activity can result in long-term increased activity and fitness (13). Other studies have examined the implementation and uptake of physical activity prescription programs, finding that they could be translated into routine clinical practice (14, 15).

Public health research has found that contact with nature is an underutilized health resource (16, 17), suggesting an opportunity to further incorporate nature-based physical activity counseling into clinical settings that leverage park and recreation resources in order to address individual and community health through physical activity as a health behavior intervention. Several clinical trials have shown that a park prescription or nature-based physical activity prescription can lead to increases in physical activity, reductions in stress and obesity, and quality of life (18–21). While PHC providers may not universally be aware of nature-based physical activity prescriptions such as park prescriptions, one study found that nearly 82% of providers expressed interest in park prescription programs (22). There is an increased recognition that nature as medicine that can be prescribed to patients to increase health and well-being.

Despite evidence that health behavior counseling promotes physical activity and can improve nutrition, it is not widely practiced (23). Within PHC, many professionals judge their own competency as lacking when it comes to knowledgeably counseling patients on activity and physical activity program options (24). Green, Cifuentes, Glasgow, and Stange found that the successful implementation of physical activity counseling relied on teamwork, clearly defining new roles within the clinic, the routine of the patient encounter, systematically assessing health behaviors to utilize that information, community linkages, and the robustness of the clinical information systems (25).

### Use of eHealth Tools

Technology as a health promotion tool has significantly evolved over the past 15 years, leading to the emergence of eHealth tools (26, 27). In today's clinical setting, eHealth tools support a patient-centered care approach that better connects patients to their health care teams, making it easier to collect patient-specific data, analyze it, and apply tailored care embedded in the clinical environment (28). Shaw et al. developed a model of eHealth that defined three domains of eHealth: 1) using eHealth to monitor, track, and inform individual health, 2) using eHealth to support communication between the patient and health care provider, and 3) using eHealth to collect, manage, and utilize health data for customized care (28). It was argued that eHealth initiatives including elements from all three domains would be the most successful (28). Three barriers to health behavior counseling are repeatedly cited: lack of time, knowledge, and resources (15, 22, 29, 30). However, eHealth tools potentially offer effective ways to reduce these barriers and streamline the integration of health behavior counseling and the prescription of health behavior changes in the daily clinical workflow by incorporating the three domains of the eHealth model.

Incorporating eHealth technologies into health behavior interventions provide the ability to customize approaches for each patient. Goal setting theory (31) predicts that setting specific goals leads to better performance when compared to vague, non-quantitative goals, such as “Be more physically active,” and has been shown to be more effective than other approaches (32). Building upon the theoretical framework of goal setting theory, using SMART goals—the original acronym stood for Specific, Measurable, Assignable, Realistic, and Time-related (33)—can help to create challenging goals specific to the individual in order to produce optimal health behavior change outcomes. Historically, evidence from randomized trials showed that if health care providers formalized their counseling (through written prescriptions rather than providing verbal advice), patients were more likely to adhere to the recommendations (34). Patient goal setting can be done through interactive technology platforms and non-clinical members of the patient's health care team (35). A study examining the integration of a health behavior counseling intervention in an electronic health record found that a shared goal-setting tool had successful usability (36). Shared decision-making engages patient and provider in a mutual negotiation process to develop more tailored and achievable chronic disease management goals.

Park prescription programs could benefit from utilizing technology that helps to collect and track individual physical activity levels (e.g., consumer fitness trackers), while also increasing communication and goal setting around the behavior change between the patient and provider (e.g., SMS text messaging) and incorporating the use of these eHealth tools into a centralized data repository (e.g., electronic medical record and prescription app compatibility).

### Clinical Intervention Models

Success of physical activity prescription programs is often attributed to their multilevel approach to patient counseling (37, 38). Physical activity prescription programs in clinical care have successfully utilized a 5 A's approach (39) in order to provide successful, tailored patient prescriptions. The 5 A's model prompts healthcare providers to engage the patient and ensure that they provide comprehensive counseling that leads to a personalized (tailored) prescription for physical activity through five intentional steps: assess, advise, agree, assist, and arrange (39). Use of the 5 A's model is a manageable, evidence-based, health behavior intervention strategy that has the potential to improve the success of nature-based physical activity prescriptions within clinical care. The Reach, Effectiveness, Adoption, Implementation, and Maintenance (RE-AIM) framework, developed by Glasgow, Vogt, and Boles (40) provides a model for evaluating public health interventions with a lens toward real-world complex settings rather than optimized research settings. The RE-AIM framework is intended to be applied throughout all stages of the research cycle as a planning and evaluation model to answer the question, “Which complex intervention, delivered by what type of staff, will be most cost effective, under which conditions, and for what outcomes?” [(41), p. e46]. Furthermore, there is an increasing call among researchers to use technology alongside the RE-AIM model to advance physical activity intervention research (42). As such, both the 5 A's and the RE-AIM model offer established and valid ways for designing innovative park-based physical activity interventions.

The GoalRx Prescription Intervention (GPI) was designed to leverage community resources to provide tailored park prescriptions for low-income patients served by Federally Qualified Health Centers (FQHC) in rural North Carolina. These interventions were based on the 5 A's (37–39) and the RE-AIM models (40–44). Focus groups were used to guide the development of the protocol used to integrate the GPI into the clinical workflow. A park audit tool was developed and a database of local park and recreation programs and resources was created to bridge the knowledge gap between community partners and medical providers. A customized web-based application was built for pairing the parks and programs database with information from the patients' electronic health records (EHR), thus creating a tailored parks prescription based on individual factors during the pre-scheduled clinic visit.

This study protocol describes a pilot observational study that explores the use of eHealth technologies to support the creation of park prescriptions among PHC providers and the effectiveness of the park prescription to increase low-income patients' time spent in moderate-to-vigorous physical activity (MVPA), assessed by accelerometry, as part of their chronic disease management care.

### Study Objectives

The primary study objective was to investigate the effectiveness of a park prescription intervention that utilizes eHealth tools (e.g., consumer fitness trackers, SMS text messaging, and an EHR integrated provider web-based application) to increase time spent in moderate-to-vigorous physical activity as assessed by accelerometry. Secondary objectives of this study were to investigate the intervention's effectiveness for:

- improving the participants' objectively measured physical health (e.g., changes in weight, blood pressure, and if applicable, blood glucose levels);
- improving the participants' self-reported mental well-being as measured by the Patient Health Questionnaire-9 (PHQ-9);
- encouraging the participants' adherence to their tailored prescription by engaging them in self-reporting texts and receipt of motivational SMS messages; and,
- streamlining the participants' enrollment into physical activity counseling using a preferences survey in the web-based application that generates customizable prescriptions automatically for the providers.

## Methods

This study protocol is based on The Standard Protocol Items: Recommendations for Intervention Trials (SPIRIT). The SPIRIT guidance document was used as a checklist to ensure the content of this protocol covered all recommended information that supported the quality of the study (45).

### Study Design

The GPI study was designed as a 3-arm, multi-site observational study. Focus groups with patients, providers, and community health and well-ness groups were used to guide the development of the protocol used to integrate the GPI into the clinical workflow. Participants received a tailored park prescription intervention, a tailored home/indoor physical activity (PA) prescription intervention, or received a healthy eating prescription (with no PA prescription beyond standard generic PA counseling advice that is already routinely provided in PHC) as the comparison group. All participants received a Fitbit Flex 2 to measure their PA levels and received SMS text message prompts for the duration of their prescription to assess prescription compliance and motivational messaging based on their individual performance. Participants in all arms completed assessments at baseline (T0) and 3-month follow-up at completion of the intervention (T1).

### Study Setting

The GPI was conducted in FQHCs in two rural counties in North Carolina, USA. Participants were recruited face-to-face during their general well-ness visits to the FQHC clinic locations if their home zip code was also in one of the two target counties and they qualified as a low-income household (i.e., recipient of Medicaid or Medicare). Participants were recruited across three clinical sites with the assistance of two onsite behavioral health clinicians (BHCs). The outdoor, nature-based, exercise component of the intervention was conducted at local and state parks throughout the targeted counties. The home/indoor physical activity prescriptions were conducted either at the participants' homes or indoors at one of the community or commercial recreation facilities in the two counties. The amenities of these local and state parks and recreation facilities were audited and cataloged by the research team as a database for the intervention using a modified version of the validated Community Park Audit Tool that was specifically modified for the study to include a full audit protocol for indoor facilities as well as parks (46, 47).

### Eligibility Criteria

Study participants had to meet all the following requirements: (1) adult ages 18 years or older. (2) reside in either of the two selected North Carolina counties. (3) qualify for Medicaid or Medicare. (4) have a clinical diagnosis for at least one of the following: pre-diabetes, diabetes mellitus, hypertension, or have undiagnosed hypertension as determined by blood pressure thresholds. (5) willing to receive a health behavior prescription. (6) able to read and speak English, and (7) provide written informed consent. Patients were excluded from the study due to any of the following criteria: (1) unwilling to sign an informed consent form. (2) documented severe alcoholism or drug abuse that could significantly affect their ability and likelihood to comply with the study requirements. (3) female patients who were pregnant or planned to become pregnant within 6 months. (4) non-ambulatory patients and patients consulted or scheduled for surgery at the time of baseline data collection, or (5) patients planning to move from the study area during the 6 months following recruitment.

All patients seen at the three clinics for a general well-ness visit were screened for eligibility to participate in the study; interested patients were briefed at the clinic by the BHC during which the study purpose, duration, benefits, and registration into the GPI study was presented. Eligible interested patients were enrolled in the intervention by the BHC through an app enrollment screening process that included an electronic informed consent form. Assessment questions were integrated into the patient preference intake survey that the BHCs administered to screen for conditions or risk factors that required further assessment by the primary physician before engaging in a physical activity. Baseline data were gathered after enrollment.

### Interventions

Participants were instructed to follow their tailored prescription SMART goal for 3 months. Participants were provided with a printed copy of their prescription SMART goal, set up with a Fitbit Flex 2 activity tracker, and enrolled to receive SMS texts from the study team in order to assess participant compliance with the prescription and provide motivational messaging around participant adherence to their prescription SMART goal and the Fitbit protocol.

#### Group 1—Intervention Park Prescription

Participants in this group received a park prescription: a self-guided physical activity prescription for a nature-based physical activity of their choice. Participants reviewed the list of potential outdoor physical activities (e.g., biking, walking, hiking, etc.) generated from their preference selection and displayed by location (descending in order of distance from their home address) and selected a SMART goal with the mutual agreement of the BHC. Each park prescription was written as a SMART goal, making sure that the goal was specific, measurable, attainable, relevant, and timely. To this end, the following parameters of the park prescription SMART goal were determined by the BHC in consideration of the participant's interest, physical ability, and overall health needs: (1) location of the park. (2) list of days of the week patient was asked to complete a session. (3) duration of the session in minutes. (4) start date of the prescription. (5) end date of the prescription, and (6) outdoor physical activity type. This resulted in a tailored prescription that read: “Do <outdoor physical activity type> for <duration per session> minutes on <list of days of the week> each week at <outdoor location> until <end date>.”

#### Group 2—Intervention Indoor Physical Activity Prescription

Participants interested in a home/indoor physical activity prescription could opt for an activity of their choice (e.g., yoga at home, group workout class, etc.). Participants reviewed the list of potential physical activities generated from their preference selection and displayed by location. If a home/indoor physical activity prescription was selected, the following parameters were determined by the BHC in consideration of the participant's interest, physical ability, and overall health needs: (1) Location of the indoor physical activity. (2) List of days of the week patient was asked to complete a session. (3) Duration of the session in minutes. (4) Start date of the prescription. (5) End date of the prescription, and (6) Indoor physical activity type. This resulted in a tailored prescription that read: “Do <indoor physical activity type> for <duration per session> minutes on < list of days of the week> each week at <indoor location> until <end date>.”

#### Group 3—Comparison Healthy Eating Prescription

Participants in the comparison group continued with their daily physical activity routine. They were not given any physical activity prescription. Instead, participants interested in a healthy eating prescription could receive a healthy eating prescription and informational handout related to (1) eating fruits and vegetables. (2) controlling portion size. (3) limiting sugary beverages. (4) eating meals at home, or (5) eating fast food (Table 1). The BHCs worked with patients to co-negotiate and select a specific healthy eating goal that was then created using the GPI app. To ensure that the automated SMS workflow read grammatically correctly, the BHCs were required to construct the prescription such that: <activity> as a verb-led statement (i.e., eat X more vegetables, or portion out X in a measuring cup), resulting in one of the following five tailored healthy eating prescriptions options.

**Table 1:** Comparison healthy eating prescriptions.

**Healthy eating topic**	**Example tailored prescription**	**Resource handout provided**
Eating fruits and vegetables	I will eat x [fruits and/or vegetables] y days per week (name specific days of the week here) for z weeks.	www.ncfamilieseatingbetter.org/EFNEP/links/handouts/Handout3-ChoosingFruitsAndVegetables.pdf
Controlling portion size	When I eat x, I will y [e.g., portion it out in a measuring cup, put it in a small bowl and put the bag back in the pantry] y days per week (name specific days of the week here) for z weeks.	www.ncfamilieseatingbetter.org/EFNEP/links/handouts/Handout8-SmartSizePortions.pdf
Limiting sugary beverages	I will drink x cups of water (If they have a specific water bottle they use, how many times will they fill it up and finish it?) y days per week (name specific days of the week here) for z weeks.	www.ncfamilieseatingbetter.org/EFNEP/links/handouts/Handout21-SmartDrinkChoices.pdf
Eating meals at home	I will make (lunch/dinner) at home x times per week (name specific days of the week here) for z weeks.	www.ncfamilieseatingbetter.org/EFNEP/links/handouts/Handout4-PlanDinner.pdf
Eating fast food	I will limit myself to x trips to fast food restaurants per (week/day) for z weeks.	www.ncfamilieseatingbetter.org/EFNEP/links/handouts/Handout20-SmartChoicesFastFood.pdf

Participants in the healthy eating comparison group also received generic advice from the BHC to be more physically active in accordance with the existing clinical standard of care. Therefore, each patient receiving a healthy eating prescription was assessed for physical activity readiness. Patients perceived to be ready to increase physical activity were told by the BHC: “In addition to your healthy eating prescription that we selected today, try to increase your physical activity to reach 150 mins/week of moderate intensity aerobic physical activity.” Participants perceived to be mostly sedentary or with compounding health concerns were told by the BHC: “In addition to your healthy eating prescription that we selected today, try to increase your physical activity by breaking up your bouts of sedentary activity.” No other discussion or information regarding physical activity was given to the patients in this comparison group during the duration of the study.

#### Fitbit Use Across All Three Groups

All study participants, regardless of study group, were provided with a Fitbit Flex 2 to measure their physical activity levels; the selected Fitbits were waterproof and could be worn during swimming or other water-based activities. Participants were required to wear the device at least 10 h while awake and asked to remove the device for charging and showering. Each participant received a paper copy of the Fitbit instructions along with verbal and written instructions to return to the clinic at the end of 2 weeks so clinic staff (either the BHC or lead nurse) could sync their Fitbit. At the baseline appointment, the BHCs were responsible for the assignment and distribution of the Fitbits to eligible study participants (Table 2). Participants were required to wear the Fitbit for a 2-week period at baseline and again at the 3-month follow-up data collection period. The first week of each 2-week period was used as a 1-week acclimatization period with the Fitbit, and the 2nd week of each period was used to provide the study data used for data analysis.

**Table 2:** BHC fitbit distribution protocol for 2-week wear periods.

**Fitbit distribution action**	**T0**	**T1**
Selected a Fitbit and recorded the Fitbit ID into the study app prior to handing the fully charged device to the participant.	X	
Demonstrated to the participant how to charge the device using the provided charging dongle and how to recognize when the Fitbit battery needed to be charged.	X	X
Fitted the device to the participants' non-dominant hand and explained how to operate the clasp to ensure the Fitbit was worn and not lost.	X	X
Explained to the participant that they were required to wear the device for at least 10 consecutive hours a day during their awake hours. Participants were advised that the Fitbit was water-resistant and could be worn while swimming but could be removed for showering and charging.	X	X
Reminded the participant to charge the device every four days to avoid a dead battery. Participants automatically received reminder SMS texts about this throughout their wear time.	X	X
Explained to the participant to return with the Fitbit to the clinic at the end of the 2-week wear period to return the device for syncing.	X	X
Looked up participants' recorded Fitbit ID, redistributed same Fitbit^*^ fully charged to the participant.		X

*T0, Baseline; T1, 3-month follow-up*

* *, If original Fitbit was lost or destroyed a new Fitbit was distributed and recorded in the GPI app*.

The BHCs were also responsible for reassignment and distribution of the Fitbits to study participants at the 3-month follow-up appointment. To redistribute the Fitbits for the follow-up wear period, the BHCs followed the same protocol as at baseline. In instances where a participant's original Fitbit was lost or destroyed, a new Fitbit was distributed and recorded in the GPI app. The BHCs instructed participants to return the device for syncing after the follow up 2-week wear period. As an incentive for participating in the GPI study, patients received the Fitbit to keep upon the completion of the study (or at the time they withdrew from the study). At the end of the study, participants received an informational handout detailing how to set up the Fitbit for personal use, tips for getting started with the Fitbit, and a link to the online user's manual. All study Fitbit accounts were deactivated at the end of the study period.

#### SMS Text Message Use Across All Three Groups

The GPI app integrated automated SMS text messaging prompts for the duration of the participants' prescriptions (see Supplementary Material for specific messages). Participants were automatically enrolled in an interactive text messaging campaign as part of the SMART goal intervention. Participants received an initial message that notified them of being enrolled in SMS reminders for their intervention prescription. Participants could opt-out of the SMS messages at the baseline visit by declining the service with the BHC or by texting “STOP” to any message during the study.

A two-way tailored text message system was used to collect information from the participant regarding their prescription compliance, while also providing a motivational message in response to the participant's reply (e.g., “Keep it up!” or “Making change is hard, keep working toward being more active.”). Integrated theory of health behavior change (48) was used as a framework for this health intervention and the SMS component. Social cognitive theory (49, 50) constructs, such as self-efficacy, were utilized in the SMS by recommending making changes and providing positive feedback; reinforcement was the basis of the feedback response system, intended to increase the likelihood of repeated positive behavior.

**Figure 1:**
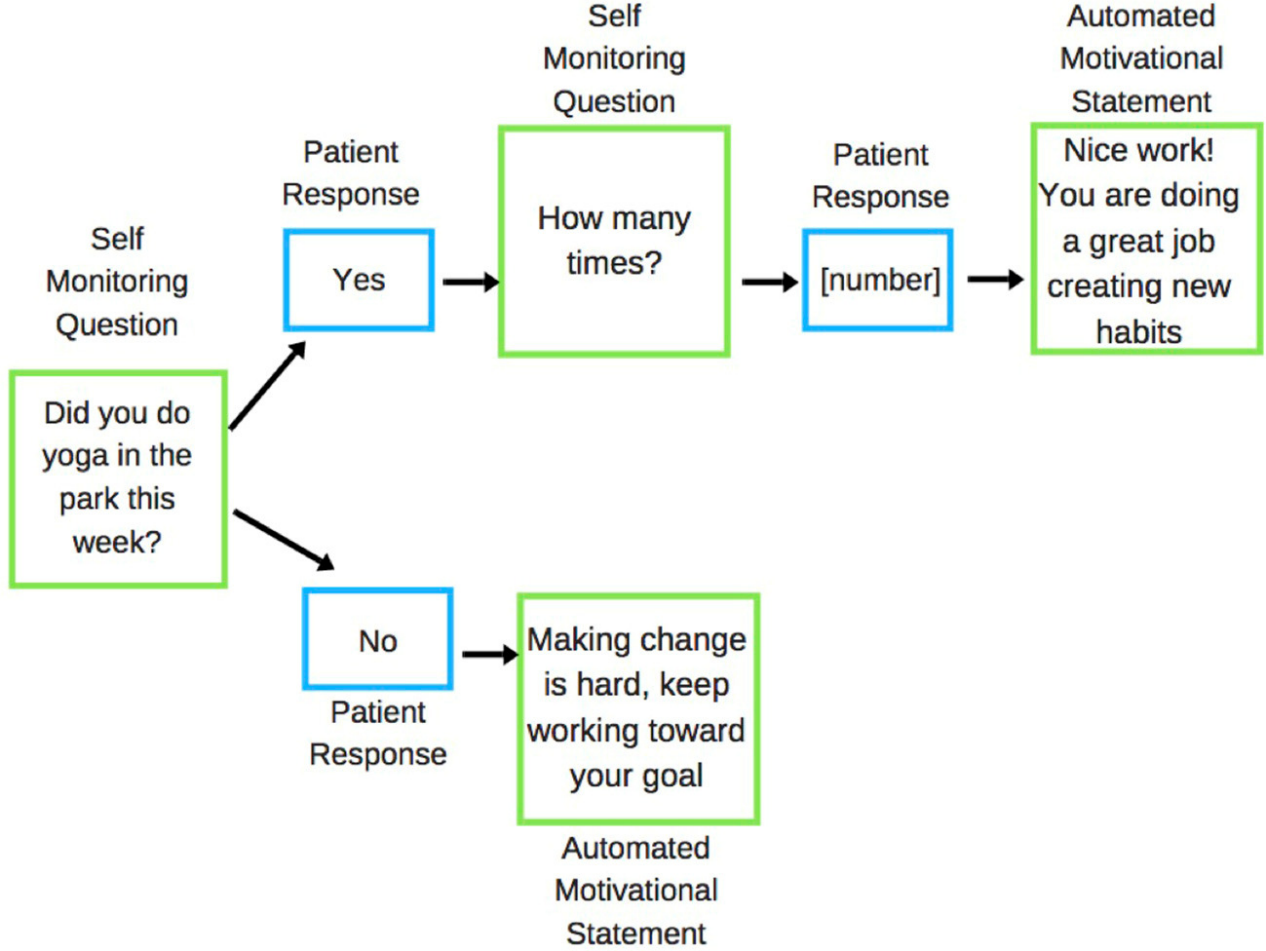
Example of the SMS feedback response system for park prescription interventions. Green boxes are messages sent to patient; blue boxes are messages received from the patient.

Self-monitoring questions were used to establish if the participant had acted upon the action specified in their prescription in the last week (e.g., attending yoga class), and if they did the specified action, then how many times they completed the action in the past week (Figure 1). Participants were asked to reply to these messages and immediately received an automated feedback response message tailored to whether or not they had met their goal (as self-reported).

### Modifications

In rare cases where a patient (*n* = 1) exhibited worsening episodes of severe or uncontrolled hypoglycemia the BHC would, when possible, modify the assigned intervention SMART goal. An unimproved health status (i.e., continued instances of severe or uncontrolled hypoglycemia) would result in the patient being withdrawn from the trial. These instances were reported as an adverse event and recorded by study staff. These cases were identified by telemedicine consults with patients between visits as well as patient visits to the FQHC in-between the scheduled 3-month encounter.

### Adherence

Each prescription group received weekly SMS messages regarding their prescription compliance. The self-monitoring questions were developed to help prompt the participant to assess their recent actions and receive motivational messages customized to their intervention goal type and recent self-reported success.

Participants also automatically received a Fitbit SMS reminder–“Avoid a low battery level. Remember to charge your Fitbit tonight.”–every four days during the 2-week Fitbit wear periods. At the end of the 2-week trial, participants received a different Fitbit SMS reminder–“It's time to return to the clinic to sync your Fitbit. Please remember to drop off your Fitbit within the next week.”–prompting them to return to the clinic to return and sync their Fitbit.

Participants received a SMS reminder for their 3-month follow-up clinical appointment with the BHC. At the end of the 3-month intervention, the SMS system was used to assess the usefulness of the text messages toward adhering to their prescription. After answering the evaluation questions, study participants received a thank you message for participating in the study.

Patients were also telephoned by either the BHC or the lead nurse to remind them of upcoming appointments and/or to reach out to patients who skipped their follow-up appointment. Participants who failed to return their Fitbits for syncing were contacted by the BHCs *via* phone as a reminder. In instances where a participant's phone number had been disconnected, or if a participant was unable to be reached by phone, a letter was sent from the clinic to the participant reminding them to return their Fitbit and asking for their help in successfully completing the remainder of the study protocol.

### Outcomes

#### Primary Outcome

The primary outcome for the study was the mean difference in weekly physical activity from baseline (T0) to post-intervention (T1) as measured by the Fitbit Flex 2 between the park intervention group and the comparison groups. Physical activity was defined by the total daily step count as measured by the Fitbit Flex 2 and the total active minutes per day. Accelerometer data is widely accepted as an objective, valid, and reliable physical activity measure for use in community trials. The accuracy of consumer wearable activity monitors has been formally assessed. While the algorithm used by Fitbit company products is proprietary, it has been used and validated in health research (51–53). The primary aim was to evaluate whether the park prescription intervention resulted in a greater change in objectively measured physical activity compared to either the home/indoor physical activity intervention group or the healthy eating comparison group, and if any physical activity changes were sustained and adopted as a lifestyle behavior for the duration of the intervention. It was hypothesized that participants in the park prescription intervention would increase their average steps/day and minutes of active time/day in T1 compared with T0 and when compared to the other intervention and comparison groups.

#### Secondary Outcomes

The secondary outcomes measures included an assessment of the relationship between the intervention and biological markers of health, including BMI, systolic and diastolic blood pressure, HbA1c or available glucose test (if applicable), and depression screen score using the PHQ-9 collected at both T0 and T1 well-ness visits to the clinic. The PHQ-9 is the major depressive disorder module of the full Patient Health Questionnaire and is a validated measure used to provisionally diagnose depression and grade severity of symptoms wherein a PHQ-9 score of 5, 10, 15, and 20 represent mild, moderate, moderately severe, and severe depression (54). The secondary outcome measures in this study were defined as the differences in mean values in the three study groups at 3-month follow-ups in mental well-being and physical health. It was anticipated that participants in the park prescription intervention group would show significant changes in these measures, while those in the home/indoor physical activity intervention and healthy eating comparison group would show no significant change. Other measures collected include potential covariates such as employment status, race/ethnicity, age, and gender.

Secondary outcomes from the SMS text messages included the total number of SMS messages sent, number of SMS messages responded to, number of SMS messaged ignored, and opt-out rate (overall and by intervention group). These measures helped assess the overall reach and impact of the feedback system. The SMS compliance messaging was used to determine prescription compliance rates. Through the self-evaluation questions, we measured if the participant followed their health behavior prescription that week, and if so, how many times. This allowed us to calculate a compliance rate per participant and per intervention goal type. In turn, this SMS data was analyzed in conjunction with the participant's tailored intervention prescription SMART goal along with the Fitbit and individual biometric data.

### Participant Timeline

**Figure 2:**
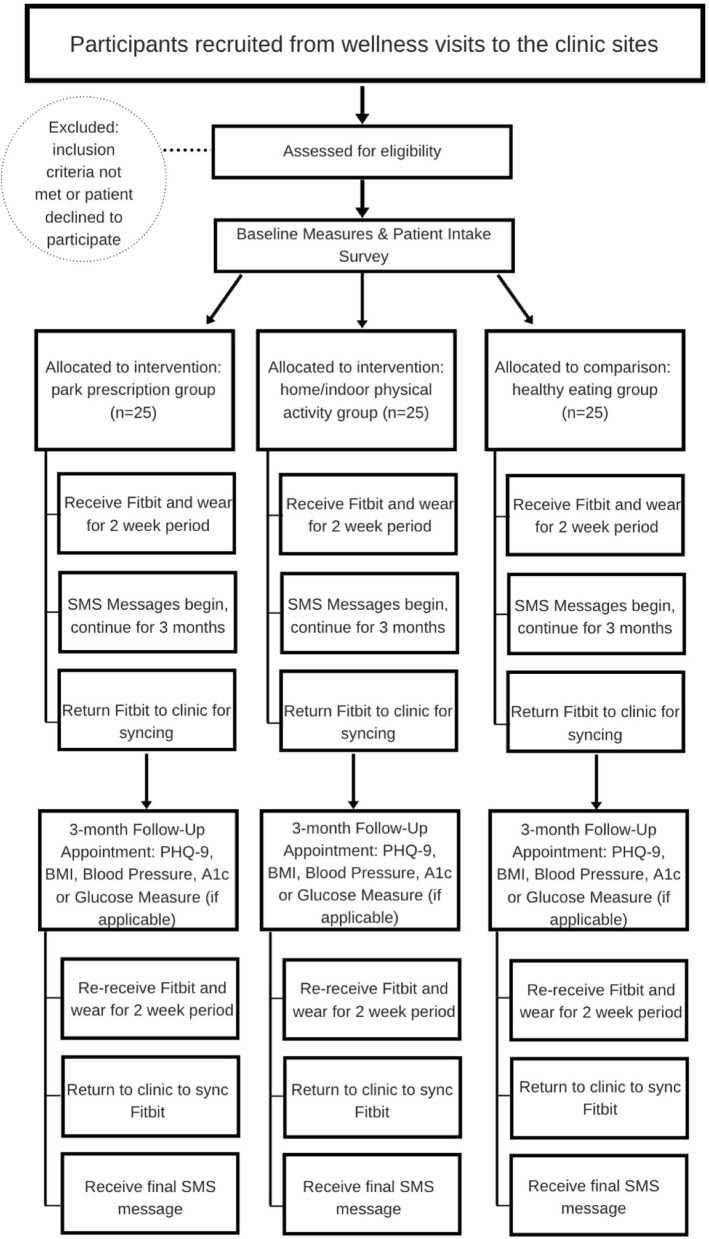
Flow of participants throughout the GPI study.

Participants were engaged in a 3-month intervention prescription SMART goal. The FQHC patients recruited for this study were already visiting the clinic for quarterly well-ness checks per conventional diabetes management. The study clinic encounters therefore, were selected to coincide with the participants regularly scheduled appointments by the clinic staff. At baseline, participants completed their well-ness visit, enrolled in the study, received their Fitbit, wore the Fitbit for 2-weeks, and then returned the Fitbit to the clinic for syncing. Patients also began receiving the SMS messages that would be sent throughout the entire study period and acting upon their tailored prescription SMART goal. The follow-up data collection required the participants to return to the clinic for a 3-month follow-up well-ness visit, re-receive the Fitbit, wear the Fitbit for 2-weeks, then return to the clinic to sync the Fitbit, and respond to the final SMS messages. Figure 2 illustrates the participation flow and includes the timeline of enrollment, intervention components, measures, and clinic visits for participants across all 3 months of the intervention.

### Sample Size Calculation

The sample size was estimated based on Viechtbauer et al.'s formula (55). Problems with a prevalence of 5% would almost certainly be identified with a 95% confidence level in a study including 59 participants. Accounting for an anticipated drop-out rate of 20%, this yields a needed sample size of *n* = 71. We opted for an enrollment of *n* = 25 per group, or *n* = 75 participants in total.

### Recruitment

Patients who lived in either of the two targeted counties in North Carolina and were patients of the partner clinics were recruited for the GPI by the BHCs at the respective sites. Recruitment occurred with a 2-month open enrollment period beginning February 7, 2018 and ending April 6, 2018. The 3-month follow-up appointments ran May through September 2018. To increase interest in the GPI, the study team partnered with the BHCs and lead nurse to promote the study through recruitment flyers posted at each clinic and directly handing flyers to patients. Two study team members also held informational sessions for front desk staff and primary-care physicians at each clinic location to introduce them to the study and encourage them to refer patients to the study through the BHCs.

The main recruitment mechanism for the GPI study was through general well-ness visits with the BHC. During those already scheduled visits, patients were given detailed information regarding study design (e.g., SMART goal, Fitbit, and SMS), duration of the study, and an informed consent form. Prospective participants read and electronically signed an informed consent form. Only patients who opted into the study had their information accessible to the research team *via* the GPI web-based application.

A customized web-based app was developed for the GPI that integrated information about patients related to their chronic disease, physical activity preferences, and nutrition behaviors to generate multiple tailored goals for each of the three study arm groups from which one approach was selected and prescribed to the patient as part of their chronic disease management care. The GPI app paired patient health data from the FQHC's EHR with stated patient preferences and triggered app-integrated SMS motivation and compliance messaging directly to the patient.

Each participant was required to complete a modified patient preference intake survey during a consultation with the BHC. The survey was completed using the GPI app and generated tailored prescription goal options around physical activity or healthy eating. The BHC then helped the patient assess the auto-generated options and co-negotiated one health behavior prescription that the patient was interested in and willing to receive (i.e., patients self-selected into the study arm based on their own preference based on the motivational interviewing by the BHC). Prior studies have shown (56) that patients' motivation to follow treatment regimens is likely to be influenced by any preference before interventions begin. For this protocol, we took a shared decision-making approach (57) as a more collaborative approach, rather than a paternalistic approach, between clinician and patient is encouraged in behavioral health care settings. Motivational interviewing utilizes a patient-centered health coaching approach which offers advantages for promoting behavior change by empowering the individual to make decisions on how to change their own lifestyle (58–60). Motivational interviewing, based in self-determination theory, has been shown to promote behavior change by increasing the intrinsic motivation to change (61).

Each participant was given a paper print out of their prescription SMART goal, a copy of their informed consent form, Fitbit with instructions. An appointment reminder postcard was presented to the front desk during checkout to ensure that the proper follow-up appointments were scheduled within the correct timeframe. It was then taken home as a schedule reminder for the next steps of the study. Clinical protocols were developed to ensure consistent implementation by the study team and BHCs.

Adherence to all intervention components was monitored and recorded by study staff. Prior to the intervention, the BHCs participated in a training led by the lead author to familiarize them with all aspects of the study protocol, go over the details required for successfully enrolling a participant, and ensure they were comfortable with navigating and using the GPI app.

### Assignment of Interventions

Participants were self-enrolled into either a treatment or comparison prescription SMART goal on a first-served basis until the sample size was reached. Once the trial slots were filled, additional patients could receive an intervention prescription as part of their clinical health care but were not included in the study. The BHCs used the GPI app and modified patient intake form to negotiate the final intervention prescription with each participant based on their medical needs, preference, and willingness to begin changing the health behavior. Due to the nature of the intervention and study logistics, participants and study staff were not blinded to the group allocation.

### Data Collection and Analysis

#### Data Collection

Patient biometric data was pulled from the electronic medical record into the GPI app (Table 3). The BHCs used the patient intake form in the GPI app to guide a patient interview and collect data including: confirming demographic (i.e., date of birth, gender, race) and health information from the EHR (i.e., height and weight used to calculate BMI, systolic blood pressure, diastolic blood pressure, PHQ-9 score, and HbA1c or available glucose reading if applicable) measured at both baseline and the 3-month follow-up, medication compliance, diabetes management knowledge (if applicable), healthy eating preferences and interest, physical activity preferences and interest, and lifestyle questions (typical form of transportation, work and/or faith community address, awareness of disease specific programs).

**Table 3:** Patient Intake Form for GPI Study.

**Data source**	**Category**	**Form field/question**	**T0**	**T1**
EMR	Patient demographics	Date of birth	X	X
		Gender	X	X
		Race/Ethnicity	X	X
Patient Visit/EMR	Patient health indices	Height	X	X
		Weight	X	X
		Systolic blood pressure	X	X
		Diastolic blood pressure	X	X
		PHQ-9 Score	X	X
		A1C, 2-h Glucose, or Fasting Glucose (*if applicable*)	X	X
Patient guided interview	Patient lifestyle	What is your home address?	X	
		What is your work address? (*if applicable*)	X	
		Do you attend a faith community? If yes, what is the location of your faith community?	X	
		What type of transportation do you use most?	X	
		Do you self-monitor your blood pressure?	X	
		If you are prescribed medicine, do you take your medication as prescribed? If no, record reason for non-compliance	X	
		Do you know about Diabetes Prevention Program/Diabetes Self-Management Education and Support Program? (*if applicable*)	X	
Patient guided interview	Healthy eating	Which of the following do you need help with most?	X	
		How do you get most of your meals?	X	
		Can you provide the name of a friend or family member who would support your efforts to eat healthier?	X	
Patient guided interview	Physical activity	Select the MET Range appropriate for the patient.	X	
		Do you prefer activities that are indoor, outdoor or both?	X	
		Do you prefer self-guided activities, program-oriented activities, or both)?	X	
		Going forward, what type of physical activity opportunities would you be interested in doing?	X	
		Can you provide the name of a friend or family member who would support your effort to be more active?	X	
Patient guided interview	Community	Please describe other resources that you would use to support your health, if they were available in your neighborhood.	X	

*T0, Baseline; T1, 3-month follow-up*.

#### Fitbit Data Collection

The Fitbit Flex 2, a three-axis accelerometer, has capacity for 7 days of detailed minute-by-minute data storage, with daily totals saved for up to 27 days. At the end of each 2-week wear period, participants returned to the clinic to sync their Fitbit. Study team members collected the devices and manually synced all devices to the unique Fitbit accounts through a Fitbit dongle set up on a researcher's computer through the online Fitbit portal. Step and activity data were extracted from the Fitbit website *via* a third-party program called Fitabase (Small Steps Labs, LLC, San Diego, CA). All activity data were exported from Fitabase in a CSV format, allowing for easier data analysis of a fully merged dataset. The second Fitbit syncing was led by the BHCs at the clinic. BHCs installed the mobile Fitbit app onto their cell phones and used the app's Bluetooth capacity to wirelessly sync the patients' Fitbits. BHCs were given access to the Fitabase platform where they confirmed each Fitbit was successfully synced prior to giving the participant materials instructing them on how to convert the Fitbit into a personal account.

To reduce intra-participant variability, the same Fitbit device was distributed to the same participant throughout the study. To minimize the chance of the Fitbit device being lost or damaged between the data collection periods, the study staff collected the devices after the baseline 2-week wear period and redistributed them at the beginning of the 3-month follow-up data collection period. In the event that a device was lost, damaged, or broken during the study, a new device was assigned, and if available, the 1-week acclimatization periods from the original and replacement device were analyzed to assess the reliability between devices; data adjustments were applied as needed.

Even when worn correctly, the fitness trackers have limitations and may underrepresent or overrepresent the amount of physical activity performed by each participant (62). Consumer activity monitors such as the Fitbit Flex 2 have a streamlined and unobtrusive form, accommodating various clothing styles to minimize no-wear compared to less aesthetically conscious clinical grade monitors. The use of consumer activity monitors lessens the potential for participants fearing or encountering social stigma for wearing a device over several weeks.

### Data Management

All participants were given a unique identification number. This identification number was used on all measurements collected. All data was de-identified from any clinic patient number or participant identifiers using the unique identification number to assure all participants health and Fitbit data remained completely confidential and unidentifiable. All datasets were de-identified, and no names were part of the datasets. A master participant identity log only included participant phone numbers and identification numbers. This log is kept separate from the main research data under password protection and only exists to allow for the correct unification of the de-identified patient data and the SMS logs which were recorded by participant phone number. All datasets are under password protection. Datasets are stored on a secure computer at the authors' university. All data was held in strict confidence per Institutional Review Board policies.

#### Participant Biometric Data

The GPI app integrates the participant's EHR along with the selected intervention prescription SMART goal and assigned Fitbit in one comprehensive study record. Secure download of the participant biometric data was ensured using a two-factor authentication log in for study team members with an authorized account. All log-ins and downloads within the app are recorded and monitored to ensure that the security protocol has not been broken, in accordance with HIPAA protection guidelines for electronic datasets.

#### Fitbit Data

Prior to distribution of the Fitbits, a unique study ID was assigned to each Fitbit device. Each ID was used to create a separate online account through the Fitbit website (63). Device set-up was simplified by associating all 75 individual Fitbit accounts with one email address. By adding a “+” and the device's study ID to the original email address (GPIFitbit@gmail.com), 75 individual email addresses (GPIFitbit+12345@gmail.com) were recognized by the Fitbit site while Google's Gmail, which does not recognize the “+” or the numbers that follow the “+,” viewed the 75 addresses as the same and simultaneously linked them all to a single email address. The research team used these accounts to generate access to all devices *via* Fitabase and facilitate the mass export of the synced data.

The study team's management of the accounts on the Fitbit website ensured that participants were not able to access activity history, nutrition trackers, earn badges, or participate in social media interfaces and other online functions that could act as potential confounders. Participants were not allowed to sync their Fitbit using the mobile phone app during the study because this would require logging into the study-managed account. Additionally, the Fitbit Flex 2 does not have a digital feedback display that shows PA levels or steps taken, serving to further reduce the likelihood for confounding influences on PA levels. To ensure that the tracker lights were not influencing the results, the daily step total was set to 100,000 for each device, ensuring that only one white light was ever lit on the device.

#### SMS Data

After developing a SMS communication strategy and library of messages, we utilized Twilio (San Francisco, California), a cloud communications company that supports programmable SMS. The Twilio platform functioned as a gateway provider, integrated into the GPI app, and provided support for organizing and sending SMS messages on a predetermined schedule. Text messages were recorded in a log, allowing us to measure undelivered and unanswered texts, as well as track all sent and received messages between the system and participants.

Access to the Twilio logs was restricted to authorized research staff and required a two-factor authentication log-in. Activity logs were exported from Twilio as a CSV file. The lead author replaced the patient phone number with the patient identification key and stripped the file of the phone number prior to sharing with the study team or conducting any analysis.

### Statistical Analysis

Descriptive and inferential analyses were performed using SPSS (Version 24). Patient biometric data from the GPI study were presented, including descriptive and intent-to-treat analyses and *t*-tests of pre-post intervention changes in BMI, systolic and diastolic blood pressure, glucose measures, PHQ-9 depression score, daily step count, average active minutes, and self-reported prescription adherence. A difference in differences regression model was used to estimate the change in daily step count for patients with the Park Prescription compared with the other interventions.

#### Fitbit Data Analysis

Data were cleaned by establishing non-wear time cut points for the Fitbit. Using the timestamp within the data set, the first week of data (acclimatization period) for each of the two-time periods were examined and removed from the final data set when a full 14 days of wear were present. The data set was examined for outliers and removed. At least 5 days of valid data were required per time period for the individual Fitbit data to be included in the analysis. Valid data was defined as a 24-h period in which at least 10 h of data wear time was recorded that included both a morning and afternoon window (e.g., 2-h). Non-wear time was analyzed as a run of zero counts lasting more than 60 min (64).

Descriptive statistics were calculated for each variable, including demographic characteristics and physical activity. Analysis of Covariance with repeated measures were used to examine the results. Models were run to conduct two analyses: 1) Examining changes in the total weekly step counts during the baseline and post-intervention period; 2) Examining changes in total active minutes per week during the baseline and post-intervention period. Analysis was run both within groups (e.g., treatment and comparison) and between groups. Statistical significance was defined as a *p* value of <0.05. Tableau (Version 2021.4.4) was used to visualize the step data and create comparison graphs of the patient data between the intervention groups.

#### SMS Data Analysis

The SMS protocol included a weekly text message sent Friday at 10:00am ET, which asked participants to report how many times in the past week they followed through on their prescribed activity (e.g., How many times this week did you engage in walking at the park?). A score was assigned to each goal based on self-monitoring data from participants. A summary score was calculated for the 12 weeks, a higher score indicative of higher overall prescription adherence. Analysis of the stored SMS data logs included participant adherence in responding to the prompts [defined as the proportion of self-monitoring texts received of the number expected over the 3-month period (*n* = 12)]. Total adherence and adherence by study week was analyzed using chi-squared tests and *t*-tests.

## Discussion

This study designed and implemented a clinical protocol to deliver tailored physical activity health behavior prescriptions to assist low-income rural patients in better managing their health. Our focus was on designing a technology assisted intervention that would quickly generate tailored prescriptions based on patients' preferences and lifestyles. The National Physical Activity Plan calls for healthcare systems to increase the priority of physical activity assessment, advice, and promotion (65). The GPI protocol promotes equitable physical activity through the healthcare setting by prioritizing local recreation and community-based resources to identify free and low-cost recreation resources.

Cost and time are frequently cited barriers to engaging in physical activity (66, 67). A meta-analysis of exercise attitudes of rural Americans frequently cited the inability to afford a gym membership and difficulties with transportation to a fitness center (68). By focusing on free or low-cost local recreation options (calculated by a geographic buffer zone using the patient's home, worksite, and place of worship if appropriate) the GPI identified resources that were appealing to the patient and fit within their perceived constraints of money and time, whether that was a local park or using the at-home/neighborhood option. Green spaces, parks, and waterways provide a counter narrative to the value of gym memberships and build upon the value of health and nature that is recently prevalent in the literature (69, 70). Park prescriptions, as used in this study can help support health behavior changes. It is worth noting that despite the high levels of “nature” in rural communities, low park access and PA remains a challenge and a consideration for promoting “nearby nature” at-home or in the neighborhood rather than destination parks (71–73). In contrast to much work that has emphasized generalized physical activity interventions, this protocol demonstrates the feasibility of tailored health behavior interventions and the value of creating community specific data repositories and resources that allow for quick and automated generation of tailored prescriptions. Reducing the burden on health care providers to have extensive local knowledge of resources and self-generating tailored prescriptions can increase the likelihood of preventative interventions being integrated into patient care.

As the proliferation of commercially available fitness trackers, mobile device platforms and smartwatches evolves on the market, the appeal of the devices and decreasing costs have led to more individuals self-monitoring their health behavior (74–78). While research has integrated wearable devices and SMS text messaging into health behavior interventions across a range of populations (79), little attention has been directed into the usability and acceptability of these approaches for low-income rural adult patients managing a chronic disease. This study protocol utilizes fitness trackers and SMS text messages to close the information cycle between patients receiving a prescription and whether they adhered to the prescription, providing necessary feedback to health care providers. While there were some barriers facing our target audience of low-income rural adult patients such as internet access, the low-cost of SMS messages (roughly $0.005 per message) and the affordability of fitness trackers made this technology beneficial for automating data collection and providing long-term engagement and support for health behavior interventions such as park prescription programs.

This protocol aimed to seamlessly integrate tailored park prescriptions into a patient's primary care appointment. The next steps are to test the scalability of the protocol and examine ways to ensure its cultural sensitivity for diverse populations. Furthermore, we hope to further integrate the patient specific external data sources (SMS messages, fitness tracker, continuous glucose monitors, etc.) into the electronic health record to provide more comprehensive care for patients with chronic diseases. The GPI app can connect the largely siloed existing platforms of EHR records and personal daily health activities outside of the clinic (physical activity, blood sugar, sleep). Unmanaged diabetic patients could benefit from the integration of daily physical activity and glucose monitoring logs that could be used to refine patient specific insulin formulas, helping to mitigate hypoglycemic episodes and encourage increased physical activity levels by identifying the patient's unique physiologic responses to physical activity types and the subsequent insulin sensitivity changes that lead to episodes of hypoglycemia and hyperglycemia by bridging these two different data sources together in the GPI app.

## Ethics Statement

The studies involving human participants were reviewed and approved by the Institutional Review Board at NC State University. The patients/participants provided their written informed consent to participate in this study.

## Author Contributions

CS conceived the study and design with guidance from JB, GB, JH, and MF. CS and JB led data collection and onsite trainings. CS led the analysis and led the manuscript writing. All authors contributed to the interpretation of the results, provided critical feedback and helped shape the research, analysis, and manuscript. All authors contributed to the article and approved the submitted version.

## Funding

This research was supported by the Grant or Cooperative Agreement Number, U58DP005468, funded by the Centers for Disease Control and Prevention. Its contents are solely the responsibility of the authors and do not necessarily represent the official views of the Centers for Disease Control and Prevention or the Department of Health and Human Services.

## Conflict of Interest

This project was funded by the North Carolina Division of Public Health's Community and Clinical Connections for Prevention and Health Branch, in collaboration with Goshen Medical Center, Inc. Representatives from the North Carolina Division of Public Health's Community and Clinical Connections for Prevention and Health Branch and Goshen Medical Center, Inc. were involved in the conceptualization of the study and had some input into the design. All other aspects of the study were managed by the research team at NC State University and Duke University along with the BHCs. The handling editor declared a past collaboration with one of the author CS.

## Publisher's Note

All claims expressed in this article are solely those of the authors and do not necessarily represent those of their affiliated organizations, or those of the publisher, the editors and the reviewers. Any product that may be evaluated in this article, or claim that may be made by its manufacturer, is not guaranteed or endorsed by the publisher.
