# Supplementary material for: Prescribing Time in Nature for Human Health and Well-Being: Study Protocol for Tailored Park Prescriptions
Source: Front Digit Health. 2022 Jul 19;4:932533. doi: 10.3389/fdgth.2022.932533 (PMC9343582; doi:10.3389/fdgth.2022.932533)
Supplement: Supplementary file 1 [file Data_Sheet_1.docx]

**Supplementary File: SMS Protocol Workflow by Intervention Group**

**Healthy Eating Comparison Group Participants Workflow**

**Fitbit Reminder for Healthy Eating Comparison Group**

This workflow sends a reminder every four days for two weeks to help remind the patient to charge their Fitbit. This will initialize after the first patient office visit and at the three-month follow-up appointment.

*Inputs*

- end_dateFitbit – Two-week end date after which no more reminders are sent

*Initialization*

This occurs after the BHC configures the tailored prescription parameters at the end of the patient intervention if the patient opts-in to receive text messages.

"Remember to wear your Fitbit for the next two weeks and charge your Fitbit every 4 days."

Repeat Every Four Days at 6pm Until End *(This will be a two-week period from the date of the tailored prescription creation)*

Message "Avoid a low battery level. Remember to charge your Fitbit tonight."

Responses

If <today> >= <end dateFitbit>:

End process

Send once 15 days after the tailored prescription parameters are generated.

Message "It’s time to return to the clinic to sync your Fitbit. Please remember to drop off your Fitbit within the next week.”

Responses

If <today> >= <end dateFitbit>:

End process

**Study Follow Up Messages for Healthy Eating Comparison Group**

This workflow sends a message at the end of the three-month intervention after they have completed their second two-week Fitbit trial. This is sent 3 months and 3 weeks after the initial tailored prescription initiation.

*Inputs*

- end_date – A date after which no more texts are sent

Repeat Every Friday at 10am Until End

Message “Did receiving the text messages help you towards your goal to eat healthier?" (choices: Yes/No)

Responses

If receive “Yes” or “No”:

- If receive “Yes or No” Send: "Thank you for participating in our Thinking Outside the Pillbox Study. We hope that your healthy eating prescription has helped you reach a healthier you!”

If <today> >= <end date>:

End process

**Home/Indoor Physical Activity Intervention Group Participants Workflow**

This workflow sends a reminder once a week to check if the patient enrolled in the program type. Then sends a reminder once a week to check if the patient has started attending the program type. Then sends a reminder once a week to check if the patient participated in the program type that past week.

*Inputs*

- Friend-name of support person listed in interview
- name - name of physical activity program coordinator
- number - phone number of contact
- program type - the name of the program
- end_date - date after which no more reminders are sent

*Initialization*

This occurs after the BHC configures the tailored prescription at the end of the patient intervention if the patient opts-in to receive text messages.

"You have been signed up for reminders to enroll in <program type>"

"Remember to contact <name> at <number> to sign up."

*Repeat Every Friday at 10am Until Receive YES response or END Process*

Message

"Have you contacted <name> at <number> to enroll in <program type>? (choices: Yes/No)”

Responses

If receive “Yes”:

1. Send: "Way to go! <program type> is a great way to be active."

If receive “No”:

- Send: "Remember to do so as soon as you can!"

If <today> >= <end date>:

- End process

*Repeat Every Friday at 10am Until Yes Response or End Process*

Message

"Have you started attending <program type>? (choices: Yes/No)”

Responses

If receive “Yes”:

- Send: “Did you attend <program type> this week?” (choices: Yes/No)

If receive “Yes:

Send: “Keep it up! Making changes can be hard, you are doing great!”

If receive “No”:

Send: “Start with small changes and build up. There is work to be done to reach your goal, but you can do it!”

If receive “No”:

- Send: "Remember to do so as soon as you can!"

If <today> >= <end date>:

- End process

*Repeat Every Friday at 10am Until Yes Response or End Process*

Message

"Did you attend <program type> this week? (choices: Yes/No)”

Responses

If receive “Yes” Send one:

- “Way to go! Stick with it.”
- “Good job! <program type> is a great way to keep moving.
- “You are doing an awesome job taking control of your health by being more active.”
- “Well done! Keep going to <program type>.”
- “Nice work! You are going a great job of being more active.”

If receive “No” Send one:

- - “Tomorrow’s a new day, try to make <program type> this week.”
  - “Making change is hard, keep working toward being more active.”
  - “Not every day is perfect, commit to making tomorrow a day of change.”
  - “Struggling to stay active with <program type>? Try reaching out to <friend> for support.”
  - “Don’t let last week define you! Recommit to attending <program type> this week.”
  - “Each day is a new chance to make a better choice for your health. Decide to make time for <program type>.”

If <today> >= <end date>:

- End process

**Fitbit Reminder for Home/Indoor Physical Activity Intervention Group**

This workflow sends one reminder every four days for two weeks to help remind the patient to charge their Fitbit. This should initialize after the first patient office visit and at the three-month follow-up appointment.

*Inputs*

- end_dateFitbit – Two-week end date after which no more reminders are sent

*Initialization*

This occurs after the BHC configures the tailored prescription parameters at the end of the patient intervention if the patient opts-in to receive text messages.

"Remember to wear your Fitbit for the next two weeks and charge your Fitbit every 4 days."

*Repeat Every Four Days at 6pm Until End (This will be a two-week period from the date of the tailored prescription creation)*

Message

"Avoid a low battery level. Remember to charge your Fitbit tonight."

Responses

If <today> >= <end dateFitbit>:

End process

*Send once 15 days after the tailored prescription parameters are generated.*

Message

"It’s time to return to the clinic to sync your Fitbit. Please remember to drop off your Fitbit within the next week.”

Responses

If <today> >= <end dateFitbit>:

End process

**Study Follow Up Messages for Home/Indoor Physical Activity Intervention Group**

This workflow sends a message at the end of the three-month intervention after they have completed their second two-week Fitbit trial. This is sent 3 months and 3 weeks after the initial tailored prescription initiation.

*Inputs*

- program type - the name of the program
- end_date – A date after which no more texts are sent

*Repeat Every Friday at 10am Until End*

Message

“Did receiving the text messages help you towards your goal to participate in <program type>?" (choices: Yes/No)

Responses

If receive “Yes” or “No”:

- If receive “Yes or No” Send: "Thank you for participating in our Thinking Outside the Pillbox Study. We hope that your physical activity prescription has helped you reach a healthier you!”

If <today> >= <end date>:

End process

**Park Prescription Intervention Group Participants Workflow**

This workflow sends a reminder once a week to check if the patient has acted upon their park-based physical activity prescription.

*Inputs*

- physical_activity - The name of the physical activity to remind the user to do
- location - The location of the physical activity
- mins - The number of minutes to remind the user to perform the activity
- days - The days of the week to remind the user to do the activity
- end_date - A date after which no more texts are sent

*Initialization*

Message

"You have been signed up for reminders to engage in <physical activity>."

*Repeat Every Friday at 10am Until End*

Message

"Have you engaged in <physical activity> this week" (choices: Yes/No)

Response

If receive “Yes”:

- First Send: “How many times this week did you engage in <physical activity>?”
- After receiving reply response First Send: “Keep it up! Making changes can be hard, you are doing great!”

If further receive “Yes” Send one:

- “Way to go! Stick with being more active.”
- “Good job! <physical activity> is a great way to keep moving.
- “You are doing an awesome job taking control of your health by being more active.”
- “Well done! Keep going to <physical activity>.”
- “Nice work! You are going a great job of being more active.”

If receive “No”:

- First Send: “There is work to be done to reach your goal, but you can do it. Start with small changes and build up.”

If further receive “No” Send one:

- - “Tomorrow’s a new day, try to make <physical activity> a priority this week.”
  - “Making change is hard, keep working toward being more active.”
  - “Not every day is perfect, commit to making tomorrow a day of activity.”
  - “Struggling to stay active with <physical activity>? Try reaching out to <friend> for support.”
  - “Don’t let last week define you! Recommit to engaging in <physical activity> this week.”
  - “Each day is a new chance to make a better choice for your health. Decide to make time for <physical activity>.”

If <today> >= <end date>:

- End process

**Fitbit Reminder for Park Prescription Intervention Group**

This workflow sends one reminder every four days for two weeks to help remind the patient to charge their Fitbit. This should initialize after the first patient office visit and at the 3-month follow-up appointment.

*Inputs*

- end_dateFitbit – Two week end date after which no more reminders are sent

*Initialization*

This occurs after the BHC configures the tailored prescription parameters at the end of the patient intervention if the patient opts-in to receive text messages.

"Remember to wear your Fitbit for the next two weeks and charge your Fitbit every 4 days."

*Repeat Every Four Days at 6pm Until End (This will be a two-week period from the date of the tailored prescription creation)*

Message

"Avoid a low battery level. Remember to charge your Fitbit tonight."

Responses

If <today> >= <end dateFitbit>:

End process

*Send once 15 days after the tailored prescription parameters are generated.*

Message

"It’s time to return to the clinic to sync your Fitbit. Please remember to drop off your Fitbit within the next week.”

Responses

If <today> >= <end dateFitbit>:

End process

**Study Follow Up Park Prescription Intervention Group**

This workflow sends a message at the end of the three-month intervention after they have completed their second two-week Fitbit trial. This will be sent 3-months and 3-weeks after the initial prescription generation.

*Inputs*

- physical_activity - The name of the physical activity to remind the user to do
- end_date – A date after which no more texts are sent

*Repeat Every Friday at 10am Until End*

Message

“Did receiving the text messages help you towards your goal to engage in <physical activity>?" (choices: Yes/No)

Responses

If receive “Yes” or “No”:

- If receive “Yes or No” Send: "Thank you for participating in our Thinking Outside the Pillbox Study. We hope that your park-based physical activity prescription has helped you reach a healthier you!”

If <today> >= <end date>:

End process

**Generic Three-month Appointment Reminder for All Groups**

This workflow sends one message 85 days after the initial tailored prescription to remind patients of their upcoming follow up appointment at the clinic

*Inputs*

- number— Phone number for the patient’s primary clinic
- end_date – A date after which no more texts are sent

*Send 85 days after the initial tailored prescription*

Message

“It’s almost time for your three-month follow up with your health care provider at Goshen. Contact <number> to confirm your appointment.”

Responses

If <today> >= <end date>:

End process
